# Supplementary figures and images for: Nuclear response to divergent mitochondrial DNA genotypes modulates the interferon immune response
Source: PLoS One. 2020 Oct 8;15(10):e0239804. doi: 10.1371/journal.pone.0239804 (PMC7544115; doi:10.1371/journal.pone.0239804)

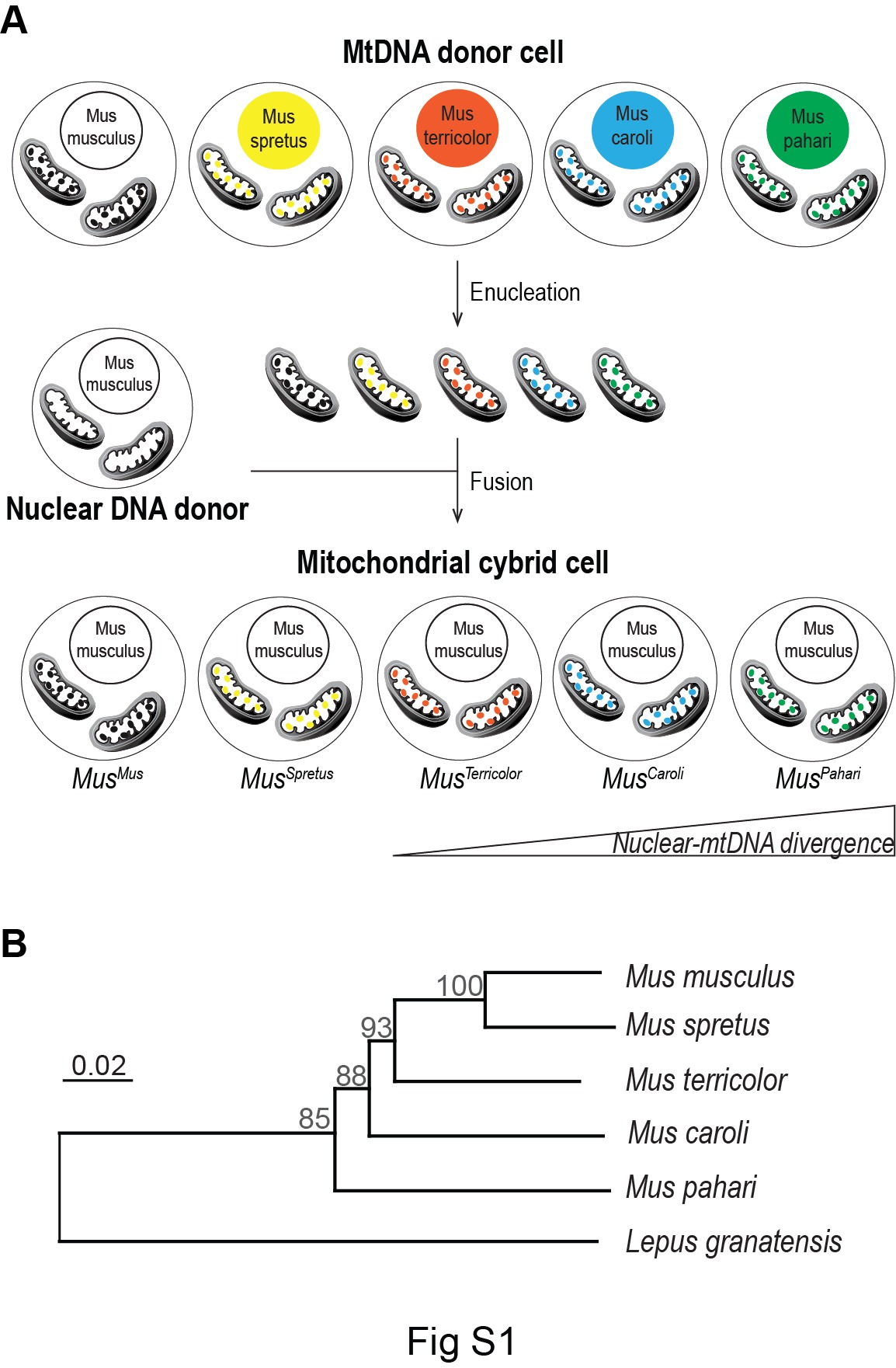

Supplement: S1 Fig — (A) Xenomitochondrial LM-thymidine kinase-negative fibroblast cybrid lines were generated by fusion of Mus musculus domesticus (Mus musculus) cells chemically devoid of mtDNA with enucleated cells harboring mtDNA from Mus musculus domesticus (MusMus; control) or exogenous mtDNA from Mus spretus (MusSpretus), Mus terricolor (MusTerricolor), Mus caroli (MusCaroli) and Mus pahari (MusPahari) to model progressively-increasing nuclear-mitochondrial genetic divergence. (B) Maximum parsimony phylogenetic tree generated from whole mtDNA sequences using Clustal Omega [45] and NJPlot software [46] show the genetic relationship between xenomitochondrial cybrid constructs. Bootstrap values are shown above the nodes (%, from 10,000 trials). Scale bar indicates percentage of substitutions per site. The tree is rooted using Lepus granatensis mtDNA sequence data (accession number NC_024042.1). (TIF) [file pone.0239804.s001.tif]

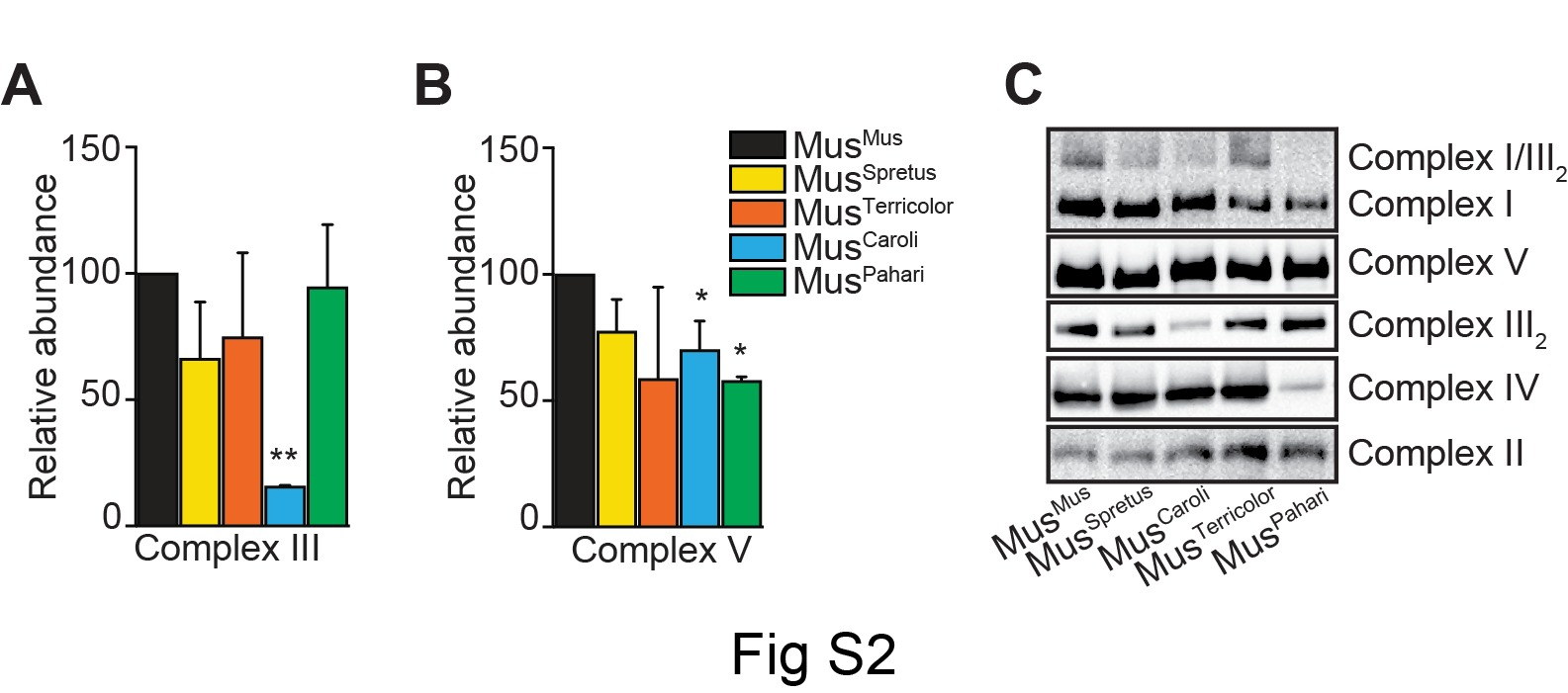

Supplement: S2 Fig — Densitometric analysis of OXPHOS complexes III (A) and V (B) by BN-PAGE show a decrease in complex III protein levels in MusCaroli and reduced complex V levels in MusCaroli and MusPahari relative to MusMus control. Protein abundance was normalized to nuclear-encoded complex II expression levels. Data presented as mean ± SD (n = 3). (C) Representative BN-PAGE immunoblot. (TIF) [file pone.0239804.s002.tif]
