# Supplementary material for: Nuclear response to divergent mitochondrial DNA genotypes modulates the interferon immune response
Source: PLoS One. 2020 Oct 8;15(10):e0239804. doi: 10.1371/journal.pone.0239804 (PMC7544115; doi:10.1371/journal.pone.0239804)
Supplement: S1 Table — (DOCX) [file pone.0239804.s003.docx]

**S1 Table.** Data used to generate high-resolution mitochondrial respiration graphs showing mean ± standard deviation.

|  | Mus^Mus^ | Mus^Spretus^ | Mus^Terricolor^ | Mus^Caroli^ | Mus^Pahari^ |
| --- | --- | --- | --- | --- | --- |
| Basal pmol/(sec*10^6^ cells) | 18.8 ± 8.0 | 46.5 ± 9.1 | 29.3 ± 11.9 | 22.5 ± 7.8 | 13.0 ± 16.0 |
| Maximal uncoupled pmol/(sec*10^6^ cells) | 127.4 ± 27.8 | 235.5 ± 25.5 | 160.2 ± 38.1 | 122.4 ± 12.3 | 92.8 ± 1.8 |
