# Supplementary material for: Nuclear response to divergent mitochondrial DNA genotypes modulates the interferon immune response
Source: PLoS One. 2020 Oct 8;15(10):e0239804. doi: 10.1371/journal.pone.0239804 (PMC7544115; doi:10.1371/journal.pone.0239804)
Supplement: S2 Table — (DOCX) [file pone.0239804.s004.docx]

**S2 Table.** Quantification of densitometric analysis from BN-PAGE immunoblots showing mean ± standard deviation relative to Mus^Mus^ control (set as 100% protein expression).

|  | Mus^Spretus^ | Mus^Terricolor^ | Mus^Caroli^ | Mus^Pahari^ |
| --- | --- | --- | --- | --- |
| Complex I | 58.7 ± 44.3 | 42.0 ± 50.4 | 21.3 ± 13.9 | 11.3 ± 9.8 |
| Complex IV | 118.4 ± 15.8 | 147.4 ± 59.0 | 119.3 ± 39.8 | 27.5 ± 13.1 |
| Complex III | 66.3 ± 22.2 | 74.8 ± 33.3 | 15.7 ± 0.3 | 94.7 ± 24.6 |
| Complex V | 77.5 ± 12.5 | 58.7 ± 36.1 | 70.1 ± 11.3 | 57.8 ± 1.5 |
