# Supplementary material for: Nuclear response to divergent mitochondrial DNA genotypes modulates the interferon immune response
Source: PLoS One. 2020 Oct 8;15(10):e0239804. doi: 10.1371/journal.pone.0239804 (PMC7544115; doi:10.1371/journal.pone.0239804)
Supplement: S3 Table — (DOCX) [file pone.0239804.s005.docx]

**S3 Table.** Data used to generate doubling time graph showing mean ± standard deviation.

|  | Mus^Mus^ | Mus^Spretus^ | Mus^Terricolor^ | Mus^Caroli^ | Mus^Pahari^ |
| --- | --- | --- | --- | --- | --- |
| Doubling time (h) | 26.0 ± 1.9 | 23.8 ± 2.9 | 22.9 ± 2.5 | 23.2 ± 2.5 | 28.7 ± 3.0 |
