# Supplementary material for: Nuclear response to divergent mitochondrial DNA genotypes modulates the interferon immune response
Source: PLoS One. 2020 Oct 8;15(10):e0239804. doi: 10.1371/journal.pone.0239804 (PMC7544115; doi:10.1371/journal.pone.0239804)
Supplement: S4 Table — (DOCX) [file pone.0239804.s006.docx]

**S4 Table.** Data used to generate superoxide production graph showing mean ± SEM.

|  | Mus^Mus^ | Mus^Spretus^ | Mus^Terricolor^ | Mus^Caroli^ | Mus^Pahari^ |
| --- | --- | --- | --- | --- | --- |
| Δ Fluorescence intensity/min | 0.43 ± 0.34 | 0.65 ± 0.40 | 0.42 ± 0.27 | 0.85 ± 0.28 | 0.50 ± 0.13 |
