# Supplementary material for: Nuclear response to divergent mitochondrial DNA genotypes modulates the interferon immune response
Source: PLoS One. 2020 Oct 8;15(10):e0239804. doi: 10.1371/journal.pone.0239804 (PMC7544115; doi:10.1371/journal.pone.0239804)
Supplement: S5 Table — (DOCX) [file pone.0239804.s007.docx]

**S5 Table.** Data used to generate mitochondrial network aspect-ratio and form factor graph showing mean ± standard deviation.

|  | Mus^Mus^ | Mus^Spretus^ | Mus^Terricolor^ | Mus^Caroli^ | Mus^Pahari^ |
| --- | --- | --- | --- | --- | --- |
| Aspect ratio | 1.8 ± 0.8 | 1.9 ± 0.9 | 1.9 ± 0.7 | 1.9 ± 0.8 | 1.8 ± 0.8 |
| Form factor | 0.8 ± 0.3 | 0.8 ± 0.3 | 0.8 ± 0.4 | 0.7 ± 0.4 | 0.9 ± 0.4 |
