# Supplementary material for: Nuclear response to divergent mitochondrial DNA genotypes modulates the interferon immune response
Source: PLoS One. 2020 Oct 8;15(10):e0239804. doi: 10.1371/journal.pone.0239804 (PMC7544115; doi:10.1371/journal.pone.0239804)
Supplement: S6 Table — (DOCX) [file pone.0239804.s008.docx]

**S6 Table.** Data used to generate graph showing log2 RNA fold change of representative ISGs showing mean ± standard deviation.

|  | Mus^Mus^ | Mus^Spretus^ | Mus^Terricolor^ |
| --- | --- | --- | --- |
| *Ifi44* | 1.07 ± 0.1 | 35.2 ± 12.2 | 6.3 ± 2.2 |
| *Isg15* | 1.1 ± 0.1 | 12.4 ± 1.1 | 3.1 ± 0.6 |
| *Irf7* | 1.07 ± 0.1 | 19.5 ± 7.2 | 4.3 ± 2.6 |
