# Supplementary material for: Nuclear response to divergent mitochondrial DNA genotypes modulates the interferon immune response
Source: PLoS One. 2020 Oct 8;15(10):e0239804. doi: 10.1371/journal.pone.0239804 (PMC7544115; doi:10.1371/journal.pone.0239804)
Supplement: S7 Table — (DOCX) [file pone.0239804.s009.docx]

**S7 Table.** ELISA data used to generate IFNα production graph showing mean ± standard deviation.

|  | Mus^Mus^ | Mus^Spretus^ | Mus^Terricolor^ | Mus^Caroli^ | Mus^Pahari^ |
| --- | --- | --- | --- | --- | --- |
| IFNα (pg/ml) | 57.3 ± 7.0 | 81.1 ± 5.0 | 77.3 ± 4.5 | 55.0 ± 3.7 | 60.4 ± 11.5 |
