# Supplementary material for: Nuclear response to divergent mitochondrial DNA genotypes modulates the interferon immune response
Source: PLoS One. 2020 Oct 8;15(10):e0239804. doi: 10.1371/journal.pone.0239804 (PMC7544115; doi:10.1371/journal.pone.0239804)
Supplement: S8 Table — (DOCX) [file pone.0239804.s010.docx]

**S8 Table.** Data used to generate graph showing log2 RNA fold change of representative basal ISG expression in kidney and liver showing mean ± standard deviation.

|  | Control | Xeno |
| --- | --- | --- |
| *Ifi44*  *kidney/liver* | 0.96 ± 0.08/  1.04 ± 0.05 | 0.10 ± 0.04/  0.51 ± 0.06 |
| *Irf7*  *kidney/liver* | 0.97 ± 0.09/  1.06 ± 0.34 | 0.43 ± 0.02/  0.74 ± 0.08 |
| *Isg15*  *kidney/liver* | 0.97 ± 0.1/  0.98 ± 0.03 | 0.54 ± 0.27/  0.63 ± 0.18 |
| *Stat2*  *kidney/liver* | 0.96 ± 0.1/  0.90 ± 0.07 | 0.61 ± 0.22/  0.81 ± 0.25 |
