# Supplementary material for: Nuclear response to divergent mitochondrial DNA genotypes modulates the interferon immune response
Source: PLoS One. 2020 Oct 8;15(10):e0239804. doi: 10.1371/journal.pone.0239804 (PMC7544115; doi:10.1371/journal.pone.0239804)
Supplement: S9 Table — (DOCX) [file pone.0239804.s011.docx]

**S9 Table.** Data used to generate viral titers graph showing mean ± standard deviation.

|  | Control | Xeno |
| --- | --- | --- |
| log_10_ PFU/ml | 4.7 ± 0.6 | 5.3 ± 0.6 |
