# Supplementary material for: Nuclear response to divergent mitochondrial DNA genotypes modulates the interferon immune response
Source: PLoS One. 2020 Oct 8;15(10):e0239804. doi: 10.1371/journal.pone.0239804 (PMC7544115; doi:10.1371/journal.pone.0239804)
Supplement: S10 Table — (DOCX) [file pone.0239804.s012.docx]

**S10 Table.** Data used to generate graph showing log2 RNA fold change of representative ISGs in brachial and axillary lymph nodes following HSV-1 infection, showing mean ± standard deviation.

|  | Control + HSV-1 | Xeno + HSV-1 | Control - HSV-1 |
| --- | --- | --- | --- |
| *Ifi44*  *brachial/axillary* | 0.97 ± 0.1/  1.04 ± 0.1 | 0.26 ± 0.1/  0.78 ± 0.17 | 0.001/ 0.001 |
| *Irf7*  *brachial/axillary* | 1.08 ± 0.1/  1.09 ± 0.2 | 0.63 ± 0.2/  0.5 ± 0.2 | 0.001/ 0.04 |
| *Isg15*  *brachial/axillary* | 1.05 ± 0.06/  1.06 ± 0.1 | 0.80 ± 0.3/  0.53 ± 0.2 | 0.001/ 0.23 |
| *Stat2*  *brachial/axillary* | 1.04 ± 0.07/  1.14 ± 0.1 | 0.74 ± 0.3/  0.75 ± 0.2 | 0.001/ 0.15 |
