# Supplementary material for: Nuclear response to divergent mitochondrial DNA genotypes modulates the interferon immune response
Source: PLoS One. 2020 Oct 8;15(10):e0239804. doi: 10.1371/journal.pone.0239804 (PMC7544115; doi:10.1371/journal.pone.0239804)

BN-PAGE: first Western blot
(4 min exposure)

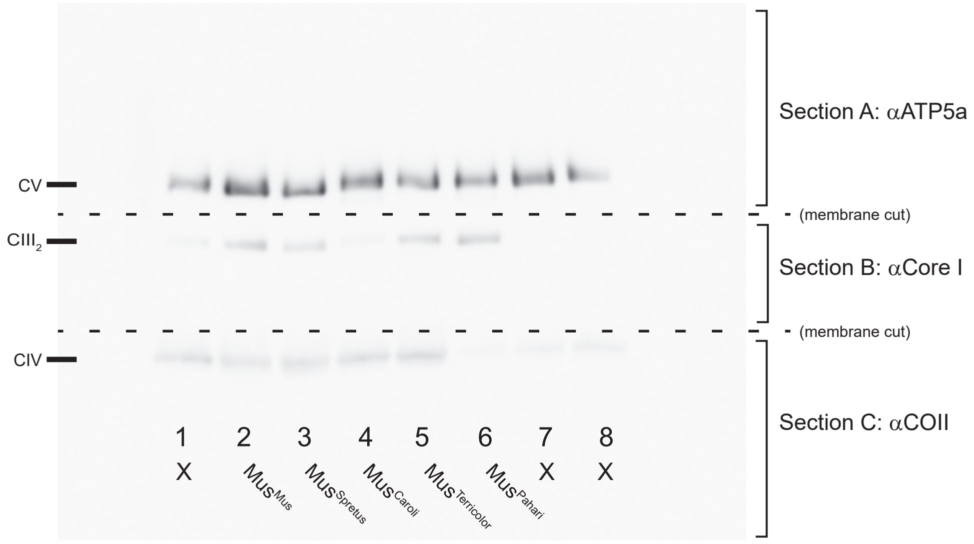

BN-PAGE: first Western blot
(16 min exposure)

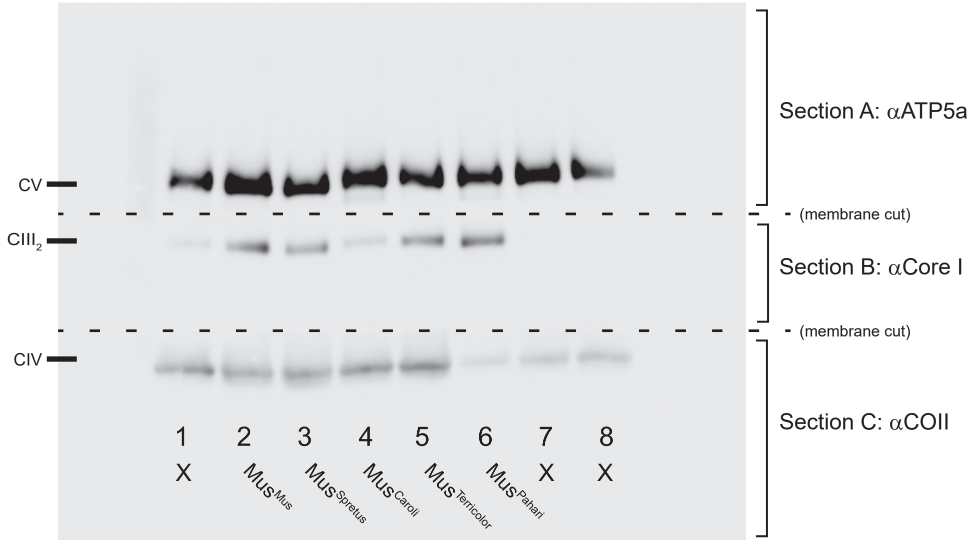

BN-PAGE: Western blot reprobe

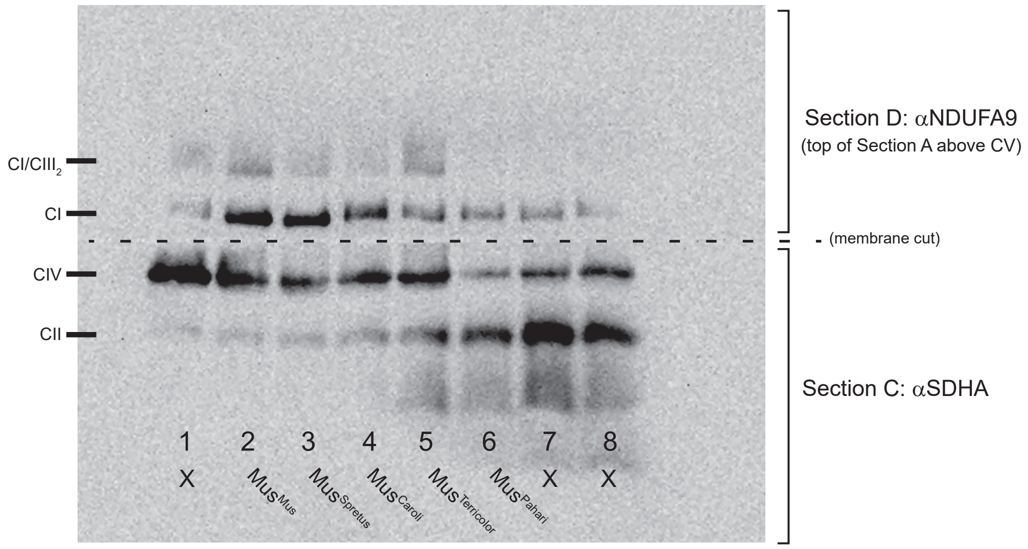

Supplement: S1 Raw images — (PDF) [file pone.0239804.s013.pdf]
